# Role of the voltage sensor module in Na_v_ domain IV on fast inactivation in sodium channelopathies: The implication of closed-state inactivation

**DOI:** 10.1080/19336950.2019.1649521

**Published:** 2019-08-05

**Authors:** Tadashi Nakajima, Yoshiaki Kaneko, Tommy Dharmawan, Masahiko Kurabayashi

**Affiliations:** Department of Cardiovascular Medicine, Gunma University Graduate School of Medicine, Maebashi, Gunma, Japan

**Keywords:** Closed-state inactivation, fast inactivation, sodium channelopathies, voltage-gated sodium channel, voltage sensor

## Abstract

The segment 4 (S4) voltage sensor in voltage-gated sodium channels (Na_v_s) have domain-specific functions, and the S4 segment in domain DIV (DIVS4) plays a key role in the activation and fast inactivation processes through the coupling of arginine residues in DIVS4 with residues of putative gating charge transfer center (pGCTC) in DIVS1-3. In addition, the first four arginine residues (R1-R4) in Na_v_ DIVS4 have position-specific functions in the fast inactivation process, and mutations in these residues are associated with diverse phenotypes of Na_v_-related diseases (sodium channelopathies). R1 and R2 mutations commonly display a delayed fast inactivation, causing a gain-of-function, whereas R3 and R4 mutations commonly display a delayed recovery from inactivation and profound use-dependent current attenuation, causing a severe loss-of-function. In contrast, mutations of residues of pGCTC in Na_v_ DIVS1-3 can also alter fast inactivation. Such alterations in fast inactivation may be caused by disrupted interactions of DIVS4 with DIVS1-3. Despite fast inactivation of Na_v_s occurs from both the open-state (open-state inactivation; OSI) and closed state (closed-state inactivation; CSI), changes in CSI have received considerably less attention than those in OSI in the pathophysiology of sodium channelopathies. CSI can be altered by mutations of arginine residues in DIVS4 and residues of pGCTC in Na_v_s, and altered CSI can be an underlying primary biophysical defect of sodium channelopathies. Therefore, CSI should receive focus in order to clarify the pathophysiology of sodium channelopathies.

## Introduction

The family of voltage-gated sodium channels (Na_v_s), encoded by the sodium voltage-gated channel alpha subunit gene (*SCNnA, n* = number), consists of nine Na_v_s (Na_v_1.1-Na_v_1.9) [1]. Na_v_s are critical determinants for action potential initiation, formation and propagation in excitable cells, including nervous system and skeletal and cardiac muscles [1,2]. The sodium flow through Na_v_s is regulated by voltage-dependent transitions between resting (closed), activated (open) and inactivated-states [2]. Transition to inactivated-states (inactivation) of Na_v_s is divided into fast, intermediate and slow components, according to the time scale of the inactivation rate. Fast inactivation greatly regulates the sodium flow through Na_v_s and can occur from both an open-state (open-state inactivation; OSI) and a closed state (closed-state inactivation; CSI) [2–4].

The Na_v_s are composed of four homologous but non-identical domains (DⅠ-DIV), and each domain contains a segment 4 (S4) voltage sensor that consists of positively charged arginine and lysine repeats. S4 segments in Na_v_s have domain-specific functions, especially in the activation and fast inactivation processes [5,6]. For OSI during strong depolarization, the S4 segments in DⅠ-DIV of Na_v_s move outward quickly and then the S4 segments in DⅢ and DIV become immobilized to promote the opening of the activation gate through a process known as electromechanical coupling, followed by the binding of the inactivation particle (conserved hydrophobic IFM motif in the cytoplasmic region linking DⅢ and DIV) to its binding sites [2,5–9]. The movement of the S4 segment in DIV (DIVS4) is particularly necessary for binding the inactivation particle to its binding sites [2,4]. On the other hand, for CSI during weak/subthreshold depolarization, only the S4 segments in DⅢ and DIV move outward without activation gate opening, but this is sufficient to allow access of the inactivation particle to its binding sites [2,4,8]. Conversely, for recovery from inactivation during repolarization, S4 segments return to their hyperpolarized/resting position, which allows the inactivation particle to unbind from its binding sites [2,4,8], and movement of DIVS4 is thought to be the rate-limiting step during repolarization [10]. Thus, DIVS4 of Na_v_s plays a pivotal role in the activation and inactivation processes.

Mutations in Na_v_s are responsible for multiple disorders (Na_v_-related diseases: sodium channelopathies) depending on the expression of each of Na_v_s on organs and its functional abnormalities [11]. Functional defects of mutations in Na_v_s can be caused by multiple mechanisms, including defective membrane trafficking, production of non-functional channels, and altered channel gating properties. Among them, altered channel gating properties, especially altered fast inactivation, can cause both a gain- and loss-of-function (and the combination of both), which can be associated with diverse phenotypes. However, although alteration of OSI of Na_v_s has received much attention, that of CSI has received considerably less attention in the pathophysiology of sodium channelopathies.

In this review, we describe the role of the voltage sensor module in Na_v_ DIV on fast inactivation, including CSI, in sodium channelopathies.

## Voltage sensor module in Na_v_ DIV

**Figure 1.**
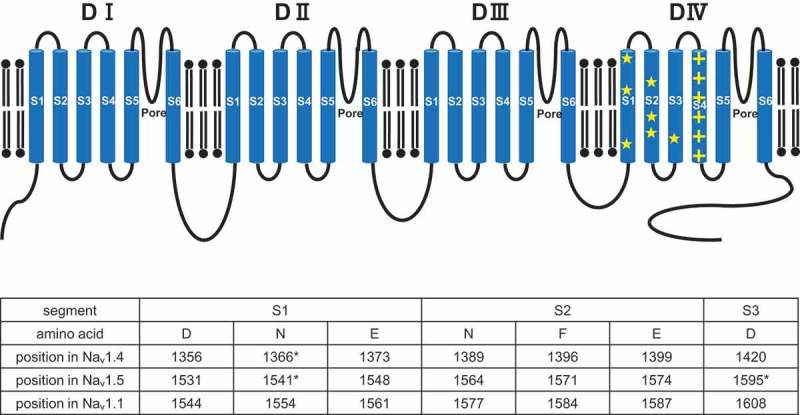
Upper panel: Predicted topology of Na_v_. Arginine residues of DIVS4 are indicated by + (yellow), and putative gating charge transfer center (pGCTC) of DIVS1-3 are indicated by ★ (yellow). Lower panel: Positions of pGCTC of DIVS4 in Na_v_1.4, Na_v_1.5 and Na_v_1.1 are listed. Asterisks indicate the positions at which disease-related mutations were functionally characterized.

Recent structural studies of Na_v_s, such as prokaryotic Na_v_Ab [12], cockroach Na_v_PaS [13], eel Na_v_1.4 [14] and human Na_v_1.4 [9], have shown that arginine residues in the S4 are in proximity to residues that may facilitate gating charge transfer (putative gating charge transfer center; pGCTC) in the S1-3 segments (Figure 1), and have established the coupling of the S4 with S1-3 in the activation and inactivation processes [15]. For example, in prokaryotic Na_v_Ab, R105 in S4 forms hydrogen bonds with N25 in S1 [12]. On the other hand, focusing on the most important domain, DIV, in the activation and inactivation processes, in Na_v_1.4, R1457 in DIVS4 (homologous residue to R105 in Na_v_Ab) forms hydrogen bonds with N1366 in DIVS1 (homologous residue to N25 in Na_v_Ab), one of the residues of pGCTC [9,16]. Since homologous residues among Na_v_s have common functional roles, the arginine residues of each DIVS4 and residues of pGCTC in DIVS1-3 play common pivotal roles in the activation and inactivation processes. Furthermore, it has become apparent that the first four arginine residues (R1-R4) in Na_v_s DIVS4 have position-specific functions in the activation and inactivation processes [9,15,17].

## Defective inactivation by mutations of arginine residues in SCN4A/Na_v_1.4 DIVS4

SCN4A/Na_v_1.4 is predominantly expressed in skeletal muscles, and mutations in Na_v_1.4 are associated with multiple skeletal muscle disorders including myotonia, paramyotonia congenita (PC), hyper or hypokalemic periodic paralysis (hyperPP or hypoPP), and congenital myasthenic syndrome (CMS). Gain-of-function of Na_v_1.4 is typically associated with myotonia, PC, hyperPP and hypoPP, and the clinical overlap of PC and hyperPP is extensive. In contrast, loss-of-function of Na_v_1.4 is typically associated with CMS [11].

Several mutations in Na_v_1.4 DIVS4 have been identified [11], and some of them have been functionally characterized (Table 1). Functional studies of R1 mutations, including R1448S, R1448H, R1448P and R1448C, associated with PC, commonly exhibited a delayed fast inactivation and hyperpolarizing shift in steady-state availability, with either a delayed or accelerated recovery from inactivation [18–24]. R2 mutations, including R1451C and R1451L, associated with PC (with or without hyperPP or hypoPP), also exhibited a delayed fast inactivation and hyperpolarizing shift in steady-state availability [25,26]. Contrary to the report of R1451L by Poulin et al. [25], Luo et al. reported that a homozygous R1451L carrier showed hypoPP and myotonia, whereas heterozygous R1451L carriers showed hyperPP and myotonia, and R1451L displayed an accelerated recovery from inactivation [26]. Intriguingly, they also reported that R1451L displayed an enhanced CSI. As described, mutations of R1 and R2 in Na_v_1.4 commonly exhibited a delayed fast inactivation, resulting in a gain-of-function of Na_v_1.4 and theoretically causing PC, although these mutations also exhibited a hyperpolarizing shift in steady-state availability that leads to a loss-of-function of Na_v_1.4. In contrast, an R3 mutation R1454W and R4 mutation R1457H, associated with CMS, commonly exhibited a hyperpolarizing shift in steady-state availability, delayed recovery from inactivation and profound use-dependent current attenuation [27,28], resulting in a severe loss-of-function of Na_v_1.4 and theoretically causing CMS, although these mutations also exhibited a delayed fast inactivation that leads to a gain-of-function of Na_v_1.4.

**Table 1.** Defective inactivation and phenotypic manifestation by mutations of arginine residues in Na_v_ DIVS4.

Gene Protein	Arginine position	Mutation	Defective inactivation	Phenotype	References
*SCN4A* Na_v_1.4	R1	R1448S	Delayed fast inactivation, hyperpolarizing shift in SSA, delayed recovery from inactivation	PC	Bendahhou et al. 1999 [18]
	R1	R1448H	Delayed fast inactivation, hyperpolarizing shift in SSA, accelerated recovery from inactivation	PC	Yang et al. 1994 [19] Chahine et al. 1994 [20] Mohammadi et al. 2003 [21]
	R1	R1448P	Delayed fast inactivation, hyperpolarizing shift in SSA,	PC	Jarecki et al. 2010 [22]
	R1	R1448L	N/A	PC	Matthews et al. 2008 [23]
	R1	R1448C	Delayed fast inactivation, hyperpolarizing shift in SSA, accelerated recovery from inactivation	PC	Yang et al. 1994 [19] Chahine et al. 1994 [20] Dice et al. 2004 [24]
	R2	R1451C	Delayed fast inactivation, hyperpolarizing shift in SSA, delayed recovery from slow inactivation,	hypoPP	Poulin et al. 2018 [25]
	R2	R1451L	Delayed fast inactivation, hyperpolarizing shift in SSA, delayed recovery from slow inactivation,	PC, hyperPP, hypoPP	Poulin et al. 2018 [25]
	R2	R1451L	Delayed fast inactivation, hyperpolarizing shift in SSA, accelerated recovery from inactivation, ***enhanced CSI,*** ***accelerated entry into CSI***	hypoPP and myotonia (homozygous), hyperPP and myotonia (heterozygous)	Luo et al. 2018 [26]
	R3	R1454W	Delayed fast inactivation, hyperpolarizing shift in SSA, delayed recovery from inactivation, delayed entry into slow inactivation, delayed recovery from slow inactivation, profound use-dependent current attenuation	CMS	Habbout et al. 2016 [27]
	R4	R1457H	Delayed fast inactivation, hyperpolarizing shift in SSA, delayed recovery from inactivation, profound use-dependent current attenuation,	CMS	Arnold et al. 2015 [28]
*SCN5A* Na_v_1.5	R1	R1623Q	Delayed fast inactivation	LQTS	Kambouris et al. 1998 [38] Makita et al. 1998 [39]
	R1	R1623L	N/A	LQTS	Splawski et al. 2000 [35]
	R2	R1626H	N/A	LQTS	Kapplinger et al. 2009 [36]
	R2	R1626H	Delayed fast inactivation, hyperpolarizing shift in SSA	AF	Olesen et al. 2012 [41]
	R2	R1626P	Delayed fast inactivation, hyperpolarizing shift in SSA	LQTS	Ruan et al. 2007 [40]
	R3	R1629G	N/A	BrS	Amin et al. 2009 [37]
	R3	R1629Q	Accelerated fast inactivation, hyperpolarizing shift in SSA, delayed recovery from inactivation, enhanced intermediate inactivation	BrS	Zeng et al. 2013 [42]
	R4	R1632H	Delayed fast inactivation, hyperpolarizing shift in SSA, delayed recovery from inactivation,	SSS	Benson et al. 2003 [31] Gui et al. 2010 [43]
	R4	R1632C	Hyperpolarizing shift in SSA, delayed recovery from inactivation, profound use-dependent current attenuation, ***enhanced CSI,*** ***accelerated entry into CSI,*** ***delayed recovery from CSI***	BrS, SSS, SVT	Nakajima et al. 2015 [34] Dharmawan et al. 2019 [57]
*SCN1A* Na_v_1.1	R1	R1636Q	N/A	LGS	Harkin et al. 2007 [47]
	R1	R1636Q	N/A	DS	Heron et al. 2010 [48]
	R2	R1639G	N/A	DS	Depienne et al. 2009 [49]
	R3	R1642S	N/A	DS	Huang et al. 2017 [11]
	R3	R1642M	N/A	DS	Lee et al. 2015 [50]
	R4	R1645Q	N/A	DS	Heron et al. 2010 [48]
	R5	R1648H	Delayed fast inactivation	GEFS+	Lossin et al. 2002 [46]
	R5	R1648H	Accelerated recovery from inactivation, decreased use-dependent current attenuation	GEFS+2	Spampanato et al. 2001 [44]
*SCN2A* Na_v_1.2	R1	R1626Q	N/A	BFIS3	Soden et al. 2014 [51]
	R2	R1629L	N/A	OS	Nakamura et al. 2013 [52]
*SCN8A* Na_v_1.6	R1	R1617Q	Delayed fast inactivation, depolarizing shift in SSA	EIEE13	Wagnon et al. 2016 [53]

AF, atrial fibrillation; BFIS3, benign familial infantile seizure 3; BrS, Brugada syndrome; CMS, congenital myasthenic syndrome; CSI, closed-state inactivation; DS, Dravet syndrome; EIEE13, early infantile epileptic encephalopathy type 13; GEFS+, generalized epilepsy with febrile seizures plus; GEFS+2, generalized epilepsy with febrile seizures plus type 2; HyperPP, hyperkalemic periodic paralysis; HypoPP, hypokalemic periodic paralysis; LGS, Lennox-Gastaut syndrome; LQTS, long QT syndrome; N/A, not available; OS, Ohtahara syndrome; PC, paramyotonia congenita; R1-R5, first five arginine residues (R1, R2, R3, R4, and R5) in domain IV-segment 4; SSA, steady-state availability; SSS, sick sinus syndrome; SVT, supraventricular tachyarrhythmia. Descriptions of closed-state inactivation are written in bold and italic.

Intriguingly, structural studies demonstrated that R1451 (R2) in Na_v_1.4 DIVS4 coupled with E1373 (Figure 1), one of the residues of pGCTC, in DIVS1, and that R1451L disrupted its electrostatic interaction with E1373 [9,14,26]. Luo et al. hypothesized that the R1451L mutation may destabilize the S4 movement that should be immobilized and allow for a quicker recovery of the S4 to the hyperpolarized/resting state, which may lead to an accelerated recovery from inactivation [26]. This concept may be supported by the fact that neutralizing or charged-reversing mutations of E1373 also accelerate the recovery from inactivation [15]. Regarding CSI, the R1451L enhanced and accelerated entry into CSI. However, recovery from CSI has not been evaluated [26].

## Defective inactivation by mutations of arginine residues in SCN5A/Na_v_1.5 DIVS4

SCN5A/Na_v_1.5 is predominantly expressed in cardiac muscles and the His-Purkinje conduction system. Gain-of-function of Na_v_1.5 is typically associated with type-3 long QT syndrome (LQT3) [29]. In contrast, loss-of-function of Na_v_1.5 can be associated with Brugada syndrome (BrS) [30], sick sinus syndrome (SSS) [31], atrioventricular conduction block [32] and supraventricular tachyarrhythmias (SVTs) [33,34], and these arrhythmic phenotypes can overlap with a single *SCN5A* mutation.

Several mutations in Na_v_1.5 DIVS4 have been identified [11,35–37], and some of them have been functionally characterized (Table 1). Mutations of R1 and R2, R1623Q (R1) and R1626P (R2), commonly exhibited a delayed fast inactivation, which leads to a gain-of-function of Na_v_1.5 and can be associated with LQT3 [38–41]. In contrast, mutations of R3 and R4, such as R1629Q (R3), R1632H (R4) and R1632C (R4), commonly exhibited a marked hyperpolarizing shift in steady-state availability, delayed recovery from inactivation and profound use-dependent current attenuation, resulting in a severe loss-of-function of Na_v_1.5 [31,34,42,43]. These functional abnormalities can theoretically be associated with BrS with or without SSS and SVTs.

A comparison of the structural alignment of Na_v_1.5 DIVS4 with Na_v_1.4 DIVS4 revealed arginine residue-specific functional abnormalities. Of note, mutations of R1 and R2 in Na_v_1.5 or Na_v_1.4 displayed a common biophysical defect of a delayed fast inactivation, resulting in a gain-of-function of either Na_v_1.5 or Na_v_1.4. In contrast, mutation of R3 and R4 in Na_v_1.5 or Na_v_1.4 displayed common biophysical defects of a hyperpolarizing shift in steady-state availability, delayed recovery from inactivation and profound use-dependent current attenuation, resulting in a severe loss-of-function of either Na_v_1.5 or Na_v_1.4.

## Mutations of arginine residues in other Na_v_ DIVS4

SCN1A/Na_v_1.1 is predominantly expressed in neuronal cell bodies and axon initial segments in the central nervous system (CNS) [11,44]. Several mutations in Na_v_1.1 DIVS4 have been identified [11,45,46], including R1636Q (R1) [47,48], found in Lennox-Gastaut syndrome or Dravet syndrome (DS), R1639G (R2) [49], found in DS, R1642S (R3) [11], found in DS, R1642M (R3) [50], found in DS, and R1645Q (R4) [48], found in DS (Table 1). However, none of them have been functionally examined.

SCN2A/Na_v_1.2 is predominantly expressed in axons and dendrites in the CNS [11,44]. R1626Q (R1) in Na_v_1.2 [51], found in benign familial infantile seizure, and R1629L (R2) in Na_v_1.2 [52], found in Ohtahara syndrome, have been identified (Table 1). However, neither of them has been functionally examined. On the other hand, SCN8A/Na_v_1.6 is predominantly expressed in the neuronal cell bodies and proximal processes in the CNS and nodes of Ranvier in the peripheral nervous system [11,44]. R1617Q (R1) in Na_v_1.6 [53], found in early infantile epileptic encephalopathy type 13, displayed a delayed fast inactivation and depolarizing shift in steady-state availability, causing a gain-of-function of Na_v_1.6 (Table 1). Notably, the delayed fast inactivation of R1617Q (R1) in Na_v_1.6 is consistent with R1 mutations in Na_v_1.4 and Na_v_1.5 which also show a delayed fast inactivation. This fact further supports the notion that arginine residues in Na_v_s DIVS4 have position-specific functions in each of Na_v_s.

## Defective inactivation by mutations of pGCTC in Na_v_1.4 DIVS1-3

In Na_v_1.4, D1356, N1366, E1373, N1389, F1396, E1399 and D1420 in DIVS1-S3 are thought to be residues of pGCTC (Figure 1) and couple with arginine residues in DIVS4 in the activation and inactivation processes [9,15]. Among them, several substituted residues have been structurally and functionally examined (Table 2).

**Table 2.** Defective inactivation and phenotypic manifestation by mutations of putative gating charge transfer center in Na_v_ DIVS1-3.

Gene Protein	Location	Mutation	Defective inactivation	Phenotype	References
*SCN4A* Na_v_1.4	DIVS1	N1366D	Delayed fast inactivation, hyperpolarizing shift in SSA, delayed recovery from inactivation	N/A	Groome et al. 2013 [15]
	DIVS1	N1366S	Delayed fast inactivation, depolarizing shift in SSA, accelerated recovery from inactivation	PC	Ke et al. 2017 [54]
	DIVS3	N1420K	Delayed fast inactivation, hyperpolarizing shift in SSA, accelerated/delayed recovery from inactivation	N/A	Groome et al. 2013 [15]
*SCN5A* Na_v_1.5	DIVS1	N1541D	Delayed fast inactivation, hyperpolarizing shift in SSA, delayed recovery from inactivation, ***enhanced CSI,*** ***accelerated entry into CSI***	BrS, SSS, SVT	Dharmawan et al. 2019 [57]
	DIVS1	E1548K	N/A	BrS	Kapplinger et al. 2010 [55]
	DIVS2	F1571C	N/A	BrS	Kapplinger et al. 2010 [55]
	DIVS2	E1574K	N/A	BrS	Kapplinger et al. 2010 [55]
	DIVS3	D1595N	Delayed fast inactivation, depolarizing shift in SSA, delayed recovery from inactivation, enhanced slow inactivation	AVB	Wang et al. 2002 [56]
*SCN1A* Na_v_1.1	DIVS1	D1544A	N/A	DS	Huang et al. 2017 [11]
	DIVS1	D1544G	N/A	DS	Huang et al. 2017 [11]
	DIVS1	E1561K	N/A	DS	Walsh et al. 2014 [58]
	DIVS3	D1608G	N/A	DS	Huang et al. 2017 [11]
	DIVS3	D1608Y	N/A	DS	Marini et al. 2007 [59]

AVB, atrioventricular conduction block; BrS, Brugada syndrome; CSI, closed-state inactivation; DS, Dravet syndrome; N/A, not available; PC, paramyotonia congenita; SSS, sick sinus syndrome; SSA, steady-state availability; SVT, supraventricular tachyarrhythmia. N1366D and N1420K in Na_v_1.4 are listed because they are homologous residue to N1541D and D1595N in Na_v_1.5, respectively. Descriptions of closed-state inactivation are written in bold and italic.

The functional effects of substitutions of these residues in Na_v_1.4 may vary depending on not only the position but also the charge of the substituted residues. Of note, structural studies showed that N1366 in DIVS1 forms hydrogen bonds with R1457 (R4) in DIVS4, and a functional study of N1366D (replaced with a negatively charged residue) displayed a hyperpolarizing shift in steady-state availability, altered voltage-dependence of fast inactivation and delayed recovery from inactivation, causing a loss-of-function of Na_v_1.4 [9,15] (Table 2). Conversely, N1366S (replaced with an uncharged residue), found in PC, displayed a cold-induced hyperpolarizing shift in activation, depolarizing shift in steady-state availability, delayed fast inactivation and accelerated recovery from inactivation, causing a gain-of-function of Na_v_1.4 that can be associated with PC [54] (Table 2). Intriguingly, a structural study of N1366S demonstrated disrupted interactions of N1366S and an arginine residue in DIVS4 at low temperature [54]. On the other hand, a structural study showed that D1420, one of the residues of pGCTC, in Na_v_1.4 DIVS3 is in close proximity to R1635 (R5) in DIVS4, and a functional study showed that D1420K (replaced with a positively charged residue) displayed a delayed fast inactivation and altered voltage-dependence of recovery from inactivation (Table 2) [15]. These findings reconfirm the importance of the coupling of arginine residues in DIVS4 with pGCTC in DIVS1-3 in the activation and inactivation processes. Regarding CSI, none of them have been examined.

## Defective inactivation by mutations of pGCTC in Na_v_1.5 DIVS1-3

In Na_v_1.5, D1531, N1541, E1548, N1564, F1571, E1574 and D1595 in DIVS1-S3 are thought to be residues of pGCTC (Figure 1) [9,15]. Among several disease-related mutations of these residues, D1595N (D1595 is the homologous residue to D1420 in Na_v_1.4) in DIVS3, found in atrioventricular conduction block, and N1541D (N1541 is the homologous residue to N1366 in Na_v_1.4) in DIVS1, found in BrS with SSS and SVTs, have been functionally examined (Table 2) [55–57].

D1595N (replaced with an uncharged residue) displayed a delayed fast inactivation, depolarizing shift in steady-state availability, delayed recovery from inactivation and enhanced slow inactivation (Table 2) [56]. In contrast, N1541D (replaced with a negatively charged residue) displayed a hyperpolarizing shift in steady-state availability, delayed fast inactivation and delayed recovery from inactivation (Table 2) [57]. It is noteworthy that these kinetic changes resemble those of N1366D in Na_v_1.4 [15]. Furthermore, N1541D displayed an enhanced CSI and accelerated entry into CSI without alteration of recovery from CSI (Tables 2 and 3) [57].

**Table 3.** Disease-related mutations in Na_v_1.4 and Na_v_1.5 that enhance closed-state inactivation.

Gene Protein	Location	Mutation	Defective inactivation	Phenotype	References
*SCN4A* Na_v_1.4	DⅢS4 (R3)	R1135C* (rat R1128C)	Delayed fast inactivation, hyperpolarizing shift in SSA, delayed recovery from inactivation, ***enhanced CSI,*** ***delayed recovery from CSI***	hypoPP	Groome et al. 2014 [60] Groome et al. 2014 [61]
	DⅢS4 (R3)	R1135H* (rat R1128H)	Delayed fast inactivation, hyperpolarizing shift in SSA, delayed recovery from inactivation, ***enhanced CSI,*** ***delayed recovery from CSI***	hypoPP	Groome et al. 2014 [60]
	DIVS4 (R2)	R1451L	Delayed fast inactivation, hyperpolarizing shift in SSA, accelerated recovery from inactivation, ***enhanced CSI,*** ***accelerated entry into CSI***	hypoPP and myotonia (homozygous) hyperPP and myotonia (heterozygous)	Luo et al. 2018 [26]
*SCN5A* Na_v_1.5	DⅡ-DⅢ linker	E1053K	Accelerated fast inactivation, delayed recovery from inactivation, enhanced intermediate inactivation, ***enhanced CSI***	BrS	Mohler et al. 2004 [66]
	DⅢ-DIV linker	Delta KPQ	Delayed fast inactivation, ***enhanced CSI,*** ***accelerated entry into CSI***	LQTS	Viswanathan et al. 2001 [63] Chen et al. 2002 [64]
	DIVS1	N1541D	Delayed fast inactivation, hyperpolarizing shift in SSA, delayed recovery from inactivation, ***enhanced CSI,*** ***accelerated entry into CSI***	BrS, SSS, SVT	Dharmawan et al. 2019 [57]
	DIVS4 (R4)	R1632C	Hyperpolarizing shift in SSA, delayed recovery from inactivation, profound use-dependent current attenuation, ***enhanced CSI,*** ***accelerated entry into CSI,*** ***delayed recovery from CSI***	BrS, SSS, SVT	Nakajima et al. 2005 [34] Dharmawan et al. 2019 [57]
	C-terminus	1795insD	Delayed fast inactivation, delayed recovery from inactivation, profound use-dependent current attenuation, hyperpolarizing shift in SSA, enhanced slow inactivation, ***enhanced CSI,*** ***accelerated entry into CSI***	LQTS, BrS	Veldkamp et al. 2000 [67] Viswanathan et al. 2001 [63]
	C-terminus	L1825P	Delayed fast inactivation, hyperpolarizing shift in SSA ***enhanced CSI,*** ***accelerated entry into CSI***	LQTS	Makita et al. 2002 [65]

BrS, Brugada syndrome; CSI, closed-state inactivation; HyperPP, hyperkalemic periodic paralysis; HypoPP, hypokalemic periodic paralysis; LQTS, long QT syndrome; R2-4, second to fourth arginine residues (R2, R3, and R4) in domain IV-segment 4; SSA, steady-state availability; SSS, sick sinus syndrome; SVT, supraventricular tachyarrhythmia. *, Electrophysiological data were obtained from rat Na_v_1.4 R1128C or rat Na_v_1.4 R1128H. Descriptions of closed-state inactivation are written in bold and italic.

## Mutations of pGCTC in other Na_v_ DIVS1-3

In Na_v_1.1, D1544, N1554, E1561, N1577, F1584, E1587 and D1608 are thought to be residues of pGCTC (Figure 1) [9,15]. Although several disease-related mutations, such as D1544A, D1544G, E1561K, D1608G and D1608Y, of residues of pGCTC in Na_v_1.1 have been identified, none have been functionally examined (Table 2) [11,58,59]. No disease-related mutations of residues of pGCTC in other Na_v_s have been reported.

## Coupling of S4 with S1-3 in Na_v_ DIV may be implicated in the CSI process

Enhanced CSI by mutations of each Na_v_ promotes the significant loss of channel availability, which can underlie the pathophysiology of sodium channelopathies. However, in functional analyses of disease-related mutations in Na_v_s, although alteration of OSI has been analyzed in detail, that of CSI has received less attention.

The CSI of disease-related mutations in Na_v_1.4 has been the most studied among Na_v_s. Functional analyses of R1128C (R3) and R1128H (R3) mutations in rat Na_v_1.4 DⅢS4, equivalent to R1135C (R3) and R1135H (R3) mutations in human Na_v_1.4 DⅢS4 found in hypoPP, respectively, displayed an enhanced CSI, accelerated entry into CSI and delayed recovery from CSI (Table 3). It has been shown that, during fast inactivation of Na_v_1.4, DⅢS4 and DIVS4 move outward, with immobilization of the DⅢS4 coupled to the binding of inactivation particle to its receptor, and return to its hyperpolarizing/resting position during repolarization [5,10]. Taken together with the results of structure-function studies, these CSI alterations of R1128C (R3) and R1128H (R3) mutations in rat Na_v_1.4 DⅢS4 may be caused by an impaired DⅢS4 movement through disrupted native electrostatic interactions of DⅢS4 and pGCTC in DⅢS2 [60,61]. In contrast, R1451L (R2) in Na_v_1.4 DIVS4 displayed an enhanced and accelerated entry into CSI (Table 3), possibly by an impaired DIVS4 movement through disrupted interactions of R1451L and a residue (E1373) of pGCTC in DIVS1 [26], consistent with the notion that neutralization of arginines in Na_v_1.4 DIVS4 enhances CSI [62], although recovery from CSI of R1451L has not been examined. These findings suggested the involvement of coupling of DⅢS4 or DIVS4 with each pGCTC in CSI in native Na_v_1.4, and may support the notion that, during subthreshold depolarization, outward movement across the membrane and subsequent immobilization of DⅢS4 and DIVS4 of native Na_v_1.4 drives entry into CSI, and its remobilization drives recovery from CSI during repolarization [2].

Regarding Na_v_1.5, enhanced CSI and accelerated entry into CSI of several mutations, such as ΔKPQ in DⅢ-DIV linker, L1825P in C-terminus, E1053K in DⅡ-DⅢ linker and 1795insD in C-terminus, have been reported [63–67]. However, recovery from CSI has never been examined. We recently examined the CSI of N1541D (homologous residue to N1366 in Na_v_1.4), one of the residues of pGCTC, in DIVS1 and R1632C (R4) (homologous residue to R1457 in Na_v_1.4) in DIVS4 in Na_v_1.5, both of which were found in BrS with SSS and SVTs [34,57]. Both N1541D and R1632C displayed an enhanced CSI; however, the mechanisms underlying CSI were not uniform. N1541D displayed a marked acceleration of the entry into CSI without alteration of recovery from CSI, whereas R1632C displayed a slight acceleration of the entry into CSI and marked delay of recovery from CSI. These findings shed light on the mechanisms underlying CSI and the structure-function relationships of Na_v_1.5. The marked acceleration of the entry into CSI of N1541D may be caused by a voltage-dependent modification of coupling of N1541D in DIVS1 with R1632 in DIVS4 during subthreshold depolarization; N1541D markedly affects the coupling in the CSI process. In contrast, the marked delay of the recovery from CSI of R1632C may be caused by a slowed remobilization of DIVS4 to the hyperpolarized/resting position during repolarization through disrupted native electrostatic interactions of DIVS4 and DIVS1, as with the R1128C (R3) and R1128H (R3) mutations in rat Na_v_1.4 DⅢS4.

Given the resemblance of functional abnormalities among homologous residues in Na_v_s, neutralizing or charge-reversing mutations of arginine residues (R1-R4) in DIVS4 or residues of pGCTC in DIVS1-3 (such as mutations of R1457 and N1366 in Na_v_1.4, homologous residues to R1632 and N1541 in Na_v_1.5, respectively, and mutations of R1626 in Na_v_1.5, a homologous residue to R1451 in Na_v_1.4) may modify the native electrostatic interactions of DIVS4 and DIVS1-3 in the activation and inactivation processes, including CSI. Further structure-function studies will be required to prove this hypothesis. Furthermore, in addition to the importance of coupling of DIVS4 with DIVS1-3 in the CSI process, other mechanisms involved in CSI may be present and need to be elucidated. Clinically, enhanced CSI of R1632C and N1541D in Na_v_1.5 is the primary biophysical defect causing a severe loss-of-function of Na_v_1.5 and may underlie the pathophysiology of overlapping arrhythmic phenotypes [57]. In this manner, enhanced CSI of other mutations in other Na_v_s may also be an underlying factor as a primary biophysical defect in sodium channelopathies.

## Lack of gating pore currents by mutations of arginine residues in Na_v_ DIVS4

Although the arginine residues of the S4 segments in DⅢ and DIV appear to have domain-specific important roles in the fast inactivation, those in DⅠ and DⅡ do not appear to have important roles [5,6]. In addition, the role of the S4 segments in Na_v_s differs with respect to gating pore currents (or ω-currents) [68–70].

Gating pore currents were originally observed in the Shaker channel by Starace and Bezanilla [71]. Substitutions of the R1 residue of S4 in the Shaker channel conduct protons or non-selective cations directly through the voltage sensor domains (gating pore or ω-pore), not through alpha (ɑ)-pore, depending on substituted residues. It is thought that disrupted interaction of substituted arginine with pGCTC may create a water crevice spanning the membrane and open a continuous aqueous pathway [68–72]. On the other hand, in Na_v_s, gating pore currents were first observed in substitutions of R1 and R2 in Na_v_1.2 DⅡS4 [73]. Later, observations of gating pore currents spread to other Na_v_s. It is noteworthy that the gating pore currents in Na_v_s have small amplitudes (less than 1% of the ɑ-pore conductance) and are smaller than those in the voltage-gated potassium channels (K_v_s) because of the formation of ɑ-pores: K_v_s have four identical gating pores but Na_v_s have only a single gating pore in each ɑ-pore. Moreover, substitutions of the arginine residues of S4 in DIV are more resistant to the creation of gating pore currents than those in DⅠ-DⅢ, possibly because the pGCTC in DIV is spread over a larger distance [70,74,75].

Evidence is accumulating on the pathophysiological roles of gating pore currents in sodium channelopathies, although there is no apparent evidence of their pathophysiological roles in potassium channelopathies. Curiously, in Na_v_1.4, all hypoPP mutations that have been identified are missense substitutions at arginine (R1 or R2) residues of S4. More surprisingly, all mutations in DⅠ-DⅢ that have been examined, except for those in DIV, create gating pore currents that flow either protons or non-selective cations, depending on the arginine positions and substituted residues [26,70,74,75]. For example, R669H (R1) and R672H (R2) in DⅡS4 create gating pore currents that conduct protons. In contrast, R672G/S/C (R2) in DⅡS4 create gating pore currents that conduct non-selective cations. In either case, these gating pore currents may account for the paradoxical depolarization of skeletal muscles and loss of excitability in low serum potassium, resulting in paralysis, in spite of the loss-of-function of Na_v_1.4 [26,70,74,75]. Intriguingly, R1135C (R3) in Na_v_1.4 DⅢS4, which is associated with hypoPP, showed enhanced CSI over a voltage range for which this mutation increases gating pore currents. This suggests that an increase of gating pore currents at subthreshold voltages may be one of the causes for the enhancement of CSI [61].

On the other hand, in Na_v_1.5, gating pore currents are thought to be associated with a particular clinical phenotype: dilated cardiomyopathy (DCM) with cardiac arrhythmias [69,70]. Most mutations in Na_v_1.5 associated with this phenotype are located at S4 in DⅠ and DⅡ, but are not present in DIV. R219H (R1) mutation of S4 in DⅠ, which does not promote apparent alteration in Na_v_1.5, creates gating pore currents that conduct protons [68,70,76]. In contrast, R222Q (R2), R225P (R3) and R225W (R3) mutations in DⅠ and R814W (R3) mutation in DⅡ, which promote either gain- or loss-of-function of Na_v_1.5, create gating pore currents that conduct non-selective cations [69,70,77–79]. Notably, it appears that mutations of arginine residues in DIVS4 do not create gating pore currents and are not associated with DCM (Table 1). The mechanisms through which the gating pore currents of Na_v_1.4 and Na_v_1.5 contribute to the clinical phenotypes need to be clarified.

## Conclusions

S4 segments in Na_v_s have domain-specific functions in terms of fast inactivation and gating pore currents. Regarding the fast inactivation, R1-R4 mutations in Na_v_ DIVS4 have position-specific functions. R1 and R2 mutations in Na_v_ DIVS4 commonly display a delayed fast inactivation, which causes a gain-of-function of Na_v_. R3 and R4 mutations in Na_v_ DIVS4 commonly display a marked hyperpolarizing shift in steady-state availability, delayed recovery from inactivation, and profound use-dependent current attenuation, which cause a severe loss-of-function of Na_v_. Thus, R1-R4 mutations in Na_v_ DIVS4 exhibit position-specific diverse phenotypes in sodium channelopathies. Regarding the gating pore currents, mutations of arginine residues of S4 in Na_v_ DⅠ-DⅢ, but not DIV, create those, which may underlie the pathophysiology of diverse clinical phenotypes. On the other hand, mutations of residues of pGCTC in Na_v_ DIVS1-3 can also alter fast inactivation. These changes in fast inactivation of Na_v_s may be caused by disrupted interactions of DIVS4 and DIVS1-3. In addition, mutations of arginine residues in Na_v_ DIVS4 and residues of pGCTC in Na_v_ DIVS1-3 can alter not only OSI but also CSI through disrupted interactions of DIVS4 and DIVS1-3. Since altered CSI can be an underlying factor as a primary biophysical defect in sodium channelopathies, CSI should receive a greater degree of focus going forward in order to clarify the pathophysiology of sodium channelopathies.
